# Proposal of a hybrid approach for tumor progression and tumor-induced angiogenesis

**DOI:** 10.1186/s12976-015-0009-y

**Published:** 2015-07-02

**Authors:** Patricio Cumsille, Aníbal Coronel, Carlos Conca, Cristóbal Quiñinao, Carlos Escudero

**Affiliations:** Department of Basic Sciences, Faculty of Sciences, Universidad del Bío-Bío, Campus Fernando May, Av. Andrés Bello s/n, Casilla 447 Chillán, Chile; Centre for Biotechnology and Bioengineering, University of Chile, Beaucheff 851, Santiago, Chile; Group of Applied Mathematics (GMA), Chillán, Chile; Group of Investigation in Tumor Angiogenesis (GIANT), Chillán, Chile; Department of Mathematical Engineering (DIM) and Center for Mathematical Modelling (CMM), University of Chile, (UMI CNRS 2807), Beaucheff 851, Correo 3 Santiago, P.O. Box 170-3 Chile; Laboratoire Jacques-Louis Lions, Université Pierre et Marie Curie and Mathematical Neuroscience Team, CIRB, Collège de France, (UMR CNRS 7598), 4 place de Jussieu, Paris, F-75005 France; Group of Research and Innovation in Vascular Health (GRIVAS Health), Chillán, Chile

**Keywords:** Hybrid approach, Tumor progression, Mathematical modelling, Parameter estimation, Experimental design

## Abstract

One of the main challenges in cancer modelling is to improve the knowledge of tumor progression in areas related to tumor growth, tumor-induced angiogenesis and targeted therapies efficacy. For this purpose, incorporate the expertise from applied mathematicians, biologists and physicians is highly desirable. Despite the existence of a very wide range of models, involving many stages in cancer progression, few models have been proposed to take into account all relevant processes in tumor progression, in particular the effect of systemic treatments and angiogenesis. Composite biological experiments, both *in vitro* and *in vivo*, in addition with mathematical modelling can provide a better understanding of theses aspects. In this work we proposed that a rational experimental design associated with mathematical modelling could provide new insights into cancer progression. To accomplish this task, we reviewed mathematical models and cancer biology literature, describing in detail the basic principles of mathematical modelling. We also analyze how experimental data regarding tumor cells proliferation and angiogenesis *in vitro* may fit with mathematical modelling in order to reconstruct *in vivo* tumor evolution. Additionally, we explained the mathematical methodology in a comprehensible way in order to facilitate its future use by the scientific community.

## Introduction

Cancer is a large group of diseases that could affect any part of the body, characterized by abnormal cell proliferation, and an increasing migration rate that could derive in invasion and organ spreading, becoming the leading cause of death all over the world [1]. Thus, cancer is a critical societal and scientific problem. Great amounts of human and material resources are yearly spent in developed countries in attempts to understand its root causes and to develop successful prevention and treatment strategies.

Most of human cancers have acquired six basic capabilities [2]: self-sufficiency in growth signals, insensitivity to growth-inhibitory signals, programmed cell death evasion, limitless replication potential, sustained angiogenesis, and tissue invasion, which could cause metastasis. In other words, the defense mechanism preventing each of these acquired capabilities must be thwarted before cells become a malignant and invasive tumor [2].

Hence, it is necessary address new strategies for cancer understanding and treatment. For doing so, establishing correlation between observable phenomena, as well as, observing if a particular intervention produces a significant response could be very useful to state hypotheses postulating which physical processes are involved and how they interact. For example, tumor cell proliferation (observable phenomenon) can be correlated to oxygen concentration (another observable phenomenon). Experimentally speaking, if we would modify the oxygen availability to observe whether cell proliferation is significantly modified, then we could estimate the oxygen threshold over which cancer cells begin to proliferate. Biological experiments needed to test such hypotheses can be time-consuming, expensive and/or impossible with current technology. In these cases, mathematical modelling plays a key role, providing an independent check of the consistency of the hypothesis and can also improve experimental design by identifying which measurements are needed to test a particular theory, and additionally, whether new hypotheses can be established from experimental results (see the review paper by Byrne [3]). In this regard, mathematical modelling is a theoretical description of biological phenomena that may be calibrated by experimental data comparison. Moreover, by changing the parameter values of descriptive equations, the significance and functions of variables representing specific biological features can be easily tested. Then, practically all tumor growth features can be mathematically modelled, reducing the biological modelling complexity and offering a powerful tool to better understand tumor biology, facilitating drug development and also pre-clinical and clinical patient management.

In this work we describe the basic principles of mathematical modelling, as well as, suggest some experimental design in order to obtain relevant biological or clinical data necessary to estimate relevant parameters of mathematical modelling. We also define *hybrid approach* as the feedback capacity between mathematical modelling and biological experimental design which is required, as it has been shown in literature, for better understanding cancer, fitting parameters of a given model with a specific biological scenario, and for obtaining models with predictive capability. This work is not intended to deepen in the previous aspects, but it can serve as a first bridge of communication between applied mathematicians, biologist and physicians or even to be a tool to emphasize research in this field.

## Overview of tumor growth and tumor-induced angiogenesis

From a point of view of materials science, a cancerous tumor is a very complex, multiscaled material. The underlying genetic defects (nano-scale) affect cellular-level (micro-scale) properties, that in turn affect material properties at the tumor level (millimeter-scale). The larger scales may even feedback to the smaller scales. For example, a change in cellular stress may affect the type and occurrence of genetic mutations. Cancer can be studied at any of the three scales as suggested by Zheng et al. [2] and the references therein.

In this work we focus at the millimeter scale, i.e., tumor tissue level. Schematically, three successive stages can be identified in the growth of a solid tumor due to deregulation of cell division: initial *avascular growth*, *angiogenesis* and finally *vascular growth*, which in turn may induce metastatic spread. For a summary of models and main mathematical references discussed in this section, the reader is addressed to Table 1.

**Table 1:** The aspects of cancer encompassed by the mathematical models discussed in this work

Model	Avascular	Vascular	Angiogenesis	Drug
	growth	growth	delivery	
[24] first work in modelling avascular tumor growth				
[32] first work in modelling tumor angiogenesis				
[2, 10, 34, 35] works focused on				
tumor angiogenesis and invasion				
[36–42] works focused in drug				
effectiveness				
[31] review of avascular tumor				
growth				
[3, 29, 30, 33] reviews of vascular				
tumor growth				

### Overview of avascular tumor growth

During the avascular growth phase, oxygen and other nutrients are delivered to the tumor cells, and the waste products are removed from the tumor via diffusion from nearby blood vessels; under this condition the tumor cells proliferate rapidly consuming more oxygen than the host cells [4]. However, considering that healthy tissue has approximately 7 % oxygen (53 mmHg) tension and the diffusion distance of oxygen in tissue is ∼100 *μ*m [5], tumor growth is limited in size [6] and maintained during a short period of time. Under these conditions, the neoplastic compartment rapidly exceeds the diffusion distance of oxygen and becomes hypoxic. Thus, oxygen tension in a tumor can range from physiological (7 %) to severe hypoxic (<1 % oxygen) or even anoxic [5]. Moreover, the immediate molecular response to low oxygen is the hypoxia-induced factor (HIF) protein stabilization [7, 8], which in turn triggers the expression of target genes involved in hypoxia adaptation such as vascular endothelial growth factor (VEGF). In addition, under severe hypoxia, it is found surrounding areas of necrosis, which is a common characteristic of solid tumors. Nevertheless, quiescent cells generation in the periphery of culture dish was observed *in vitro* during a nutrient-deficient medium and hypoxia exposure in tumor cells [9]. Therefore schematically, in the hypoxic phase, a tumor grows within the limits of its local environment forming three characteristic layers: 1) cells towards the center, deprived of vital nutrients, will die and give rise to a necrotic core; 2) proliferating cells can be found in the outer cell layers; and 3) a layer of quiescent (or hypoxic) cells, which survive without dividing with slow metabolism, is found between the two others layers. In summary, tumor growth is severely restricted and in order to continue its developing needs to find additional nutrient sources.

### Overview of vascular tumor growth

As a result of hypoxic pressure some tumor cells secrete a number of diffusive chemical substances - called *tumor angiogenic factors* (TAF), such as VEGF, into the surrounding tissue, which encourage the body to vascularize the tumor and therefore provide new nutrients. Indeed, transition from the avascular to the vascular state, depends on the tumor ability to induce new blood vessels formation from the surrounding tissue [10]. These blood vessels sprout towards the tumor and then gradually surround and penetrate it, providing an adequate micro-circulation and blood supply. Tumor-induced angiogenesis, the process by which new blood vessels develop from an existing vasculature, through sprouting, proliferation and fusion of endothelial cells, therefore is a critical step in solid tumor growth.

Under hypoxia, cancer stem cells (CSC), that reside inside the tumor, could differentiate toward endothelial progenitor cells and mature endothelium, which in turn generates new blood vessels inside the tumor [11]. This last process is called neovasculogenesis, and in the past was thought to happen only intrauterine, but nowadays it is known that this event happens also in adulthood. In cancer field, presence of CSC has been associated with tumor recurrence, resistance to chemotherapy, tumor metastasis, and in general with poor clinical prognosis [12, 13]. Tumor-induced angiogenesis is believed to start when a small avascular tumor exceeds a critical diameter (∼2 mm), above which normal tissue vasculature is no longer able to support its growth. By the time a tumor has grown to a size whereby it can be detected by clinical means, there is a strong likelihood that it has already reached the vascular growth phase (see [10] and the references therein).

Tumor-induced angiogenesis is characterized by a chaotic tumor vessels development associated with both angiogenesis and vasculogenesis. Thus, endothelial cells proliferate and capillaries are rapidly formed allowing tumor growth, but in cancer framework theses processes are deregulated. Indeed, vessel diameter is five times bigger than in normal tissue [14], rarely differentiated into arterioles or venules, with frequently blind endings, and incomplete and abnormal endothelial cell lining [15–17]. All these abnormalities generate an irregular blood flow [5], perpetuate the intermittent low oxygen delivery, increase HIF activity and promote pro-angiogenic signals generation [18–20].

Over the past decade much work has been performed to understand the angiogenic process. For instance, it is well-known that increased density of blood vessels (the so-called “hot spots”), and high VEGF plasma levels are a powerful prognosis tool in many human tumor types [21]. In fact, the capacity to modify this process has been considered as a keystone for cancer treatment, which includes some molecules that reduce the ability of TAF, and in particular of the VEGF, to provide blood supply toward the tumor and thus controlling tumor growth. Indeed, a recent meta-analysis [22] including 24 randomized trials with 8 different types of cancers, in which the synthetic antagonist of VEGF (Bevacizumab®;) was used in combination with chemotherapy, shown a statistically significant improvement in the overall survival and progression of free cancer survival in patients who received Bevacizumab®; compared with those who did not receive this drug. These beneficial effects were more evident in patients with colon cancer and renal cell carcinoma, but less evident in those who had breast, pancreatic or prostate cancer. This suggests that despite the overall benefit of Bevacizumab®;, some patients and types of cancer are more resistant to antiangiogenic therapy. A possible explanation to this phenomenon is the presence of CSC, whose differentiation could occur independently of VEGF [23].

Altogether these last evidences indicate that angiogenesis during tumor growth is a complex process which study demands the use of many experimental approaches, and actually experimental analysis is in general expensive and/or difficult to be carried out and moreover no experiment can fully explain this process. For these reasons, mathematical modelling might theoretically combine a broad range of biological events for giving a better vision of the overall tumor progression including angiogenesis.

Since the seminal work of Greenspan [24], the mathematical modelling of avascular solid tumor growth has been rapidly expanding. Most models in this area consist of nonlinear partial differential equation systems (e.g. see References [25–28]), and may be described as a macroscopic approach. In this work we will focus on tumor-induced angiogenesis and on some of the key aspects of vascular tumor growth, so we will omit a bibliographical discussion about avascular tumor growth in this manuscript, but we refer to exceptional work [3, 29–31] and the references therein.

Modelling tumor-induced angiogenesis has a well-established history beginning with the work of Balding and McElwain [32]. These authors proposed a simple model of tumor angiogenesis to describe experiments in which tumor cells implanted in the rabbit cornea stimulated formation, growth and migration of new blood vessels from the corneal limbus to the tumor [3]. Since the pioneer work of these authors [32], much of the mathematical modelling has focused on the way in which TAF initiate and coordinate capillary growth [33]. The next phase in tumor development, namely vascular tumor growth, has received less attention than avascular growth and angiogenesis in mathematical modelling literature. See also [34, 35] for early work on vascular tumor growth and invasion.

In order to show some examples in which mathematical modelling combined with biological experiments have contributed to a better cancer understanding, we will highlight some selected results centered on targeted therapies efficacy. In the papers [36–38], the authors studied multiscale models to address questions related to prediction of chemotherapy and radiotherapy efficacy. Likewise, Lignet et al. [39] and Panovska et al. [40] developed models to investigate the combined effect of anti-proliferative therapy with anti-TAF or anti-vascular therapy. Additionally, Billy et al. [41] studied the efficacy of a new anti-angiogenesis treatment and provide some indications about the best way to optimize this cancer treatment strategy. In our very recent work [42], we dealt with tumor drug resistance modelling in hepatic gastrointestinal stromal tumor (GIST) metastases, which exhibit resistance to two standard treatments: imatinib and sunitinib. Based on an accurate analysis of medical images, we provided a patient-dependent model that reproduces qualitatively and quantitatively spatial tumor evolution, as shown the follow-up clinical data that we have carried out. Interestingly, specific aspects of tumor growth as spatial heterogeneity and treatment failures could be explained by our model. See Section Looking for a hybrid approach.

Additionally, also concerning the combination of mathematical modelling with clinical data, another recent work from our group described the basic principles of mathematical modelling, as well as, the advantages, limitations and future prospects using both oncology imaging and modelling [43]. Combination of imaging and modelling can resolve complex problems and describe many aspects of tumor growth or response to treatment, and therefore nowadays is possible to consider its clinical use in the medium term. Accordingly, in the present work we also describe some aspects of tumor growth modelling giving more details and, at the same time, we suggest some biological experiments, as well as, the way of combining them to provide new insights in tumor progression, angiogenesis and response to treatments. To do this, in the next section we focus on deterministic models which analyze tumor growth as a macroscopic mechanical process involving the whole tumor tissue rather than analyzing individual cells, in the same way as models presented so far in this section. In addition, in the Section Looking for a hybrid approach, we give some insights about how mathematical modelling should be modified for fitting with the biological scenario of a specific cancer.

## Basic principles of mathematical modelling of tumor growth

Taking into account the complexity of the processes involved in all the stages of tumor growth, it is not difficult to understand that mathematical models are to a large extent basically phenomenological and simplified compared to what is happening in a biological context. Roughly speaking, from a biological point of view two main types of modelling can be distinguished: those that only consider the tumor as a whole, and those that also consider the spatial distribution of tumor components [43].

The key challenge is to develop mathematical models for tumor progression including the aspects mentioned above, in which optimal combination of drugs is simulated in order to suggest the best protocols for improving clinical outcomes. In addition, these models may be used to shed light about the existence of new mechanisms that could explain phenomena clinically observed and may have predictive capability. To do this, parameter estimation of these models is a key issue for which performing experiments or collecting clinical data are required. Then, in this work we want to emphasize two main aspects; the first one is the mathematical modelling of tumor progression; and the second one is how data coming from experimental biology or clinical data may contribute to parameter estimation.

### Non spatially-structured models of tumor growth based on ordinary differential equations

We present below some simple models considering only the change in volume at which tumor growth occurs, without taking into account tumor environment. We focus on models based on ordinary differential equations (ODE). To do this, we borrow some ideas by Byrne [44]. See Appendix A for a more detailed discussion.

**Fig. 1:**
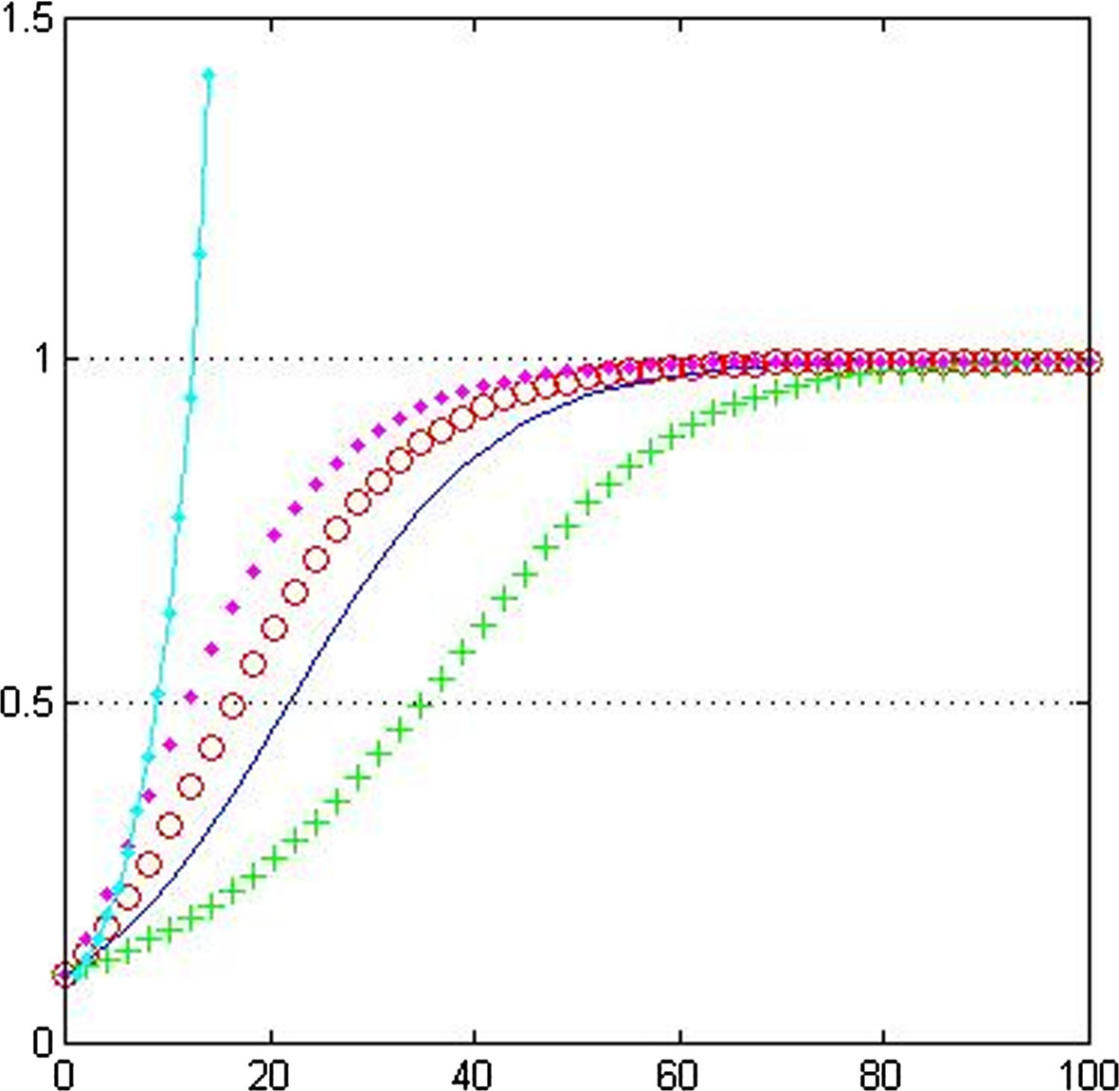
Plot of the different tumor growth laws. Exponential growth law (solid-dotted line); logistic growth (solid line); general growth law (*α*=0.5, circles and *α*=2.0, crosses); Gompertzian growth law (dots). Parameter values: *k*=0.1, *θ*=1.0, *N*_0_=0.1

One of the simplest models that can be used to describe the way in which the number of cells *N*(*t*) within a solid tumor changes over time is the exponential growth law. In this model, there are no constraints on cell growth: all nutrients and other vital growth factors are assumed to be available in abundance. In consequence, the model predicts that the population will increase exponentially, without limit. See Fig. 1.

Whilst the exponential growth law is not realistic enough, it provides an accurate description of the early stages of a tumor development. In particular, reduced growth and eventual saturation are observed when avascular tumors are grown *in vitro* or when vascular tumors develop *in vivo*. This discrepancy arises because as the tumor increases in size, competition for nutrients and other vital resources, such as space, can no longer be neglected. A simple modification of the exponential growth law which takes account of competition for resources (without specifying what those resources are) is the logistic growth law.

Whilst the logistic growth law predicts almost exponential growth of small tumors and growth saturation when the tumor reaches its carrying capacity (see Fig. 1), the symmetry of *N*(*t*) about its point of inflection means that it is not particularly flexible to fit or describe experimental data. A more general family of curves, which, depending on the choice of a parameter *α* can saturate more or less fastly than the logistic growth law, is given by

(1) $$ \frac{\mathrm{d} N}{\mathrm{d} t} = \frac k{\alpha} N \left[ 1- \left(\frac N{\theta} \right)^{\alpha} \right], \qquad \text{with} \,N(t=0)=N_{0},  $$

where *k*>0 represents the net rate at which tumor cells proliferate and *θ*>0 denotes the carrying capacity.

We remark that the logistic growth law is a special case of (1) (set *α*=1) and that the Gompertzian growth law is recovered in the limit as *α*→0^+^, which states that the net proliferation rate of tumor cells exponentially decreases with time.

In order to compare the four models presented above, we plot in Fig. 1 growth curves for each model for fixed values of the proliferation rate *k* and the carrying capacity *θ*.

These models are too simple to provide useful, reliable and reproducible information about the tumor from the phenomenological change in its volume, which varies between different tumors and patients. They may, however, be made more complex to incorporate tumor cell heterogeneity (coming from the cell cycle) or to take into account different processes such as angiogenesis; see Reference [43].

In order to get models with predictive capability an important issue is parameter estimation, which has to be performed for fitting modelling with experimental or clinical data. For example, estimating parameters *k* and *θ* for the logistic model (see (13) in Appendix A) is roughly made by minimizing the error

(2) $$  E(k,\theta)=\sum\limits_{i=1}^{M} (\hat N_{i} - N(t_{i}))^{2},  $$

where $\hat N_{i}$, *i*=1,…,*M* are *M* experimental or clinical measurements collected at different times, say *t*_*i*_ (*i*=1,…,*M*), during the tumor evolution, corresponding to the observed numbers of tumor cells at the time instants *t*_*i*_, and *N*(*t*_*i*_) are the numbers of tumor cells predicted by the model at time instants *t*_*i*_. The minimization of the error *E*(*k*,*θ*) (2) is made in this case with respect to the parameters *k* and *θ* in such a way that the responses of the model *N*(*t*_*i*_) approximately match the data $\hat {N}_{i}$. When the minimum of (2) is reached for some $(\hat {k},\hat {\theta })$, the error $E(\hat {k}, \hat {\theta })$ is called the *least-squares error* whereas $(\hat {k}, \hat {\theta })$ is known as the *least-squares estimate* of the parameter (*k*,*θ*). It is worth noting that the optimization process can be quite complicated depending on the model, the number of parameters and of the availability of data; the interested reader is referred to the textbook [45].

Classically the mathematical models used for clinical applications are based on systems of non-linear ODE, which do not consider the spatial aspect of tumor growth. Despite of it, these models have great interest in biological applications and typically they are parametrized using statistical methods and may provide a prognosis of tumor volume, but neither shape nor location of tumor can be estimated. See references [46, 47].

In order to appreciate the impact of parameter estimation in the clinical context, for example we refer to a recent meta-analysis [48] which describes natural development of meningiomas, a kind of neurological tumor. In [48], 22 studies reporting 675 patients with untreated meningiomas were found, followed by serial magnetic resonance imaging (MRI) during 5 years. From the analysis, authors show that tumors which initial diameter was >2.5 cm exhibit a linear growth rate >10 *%* per year, which leads to the highest risk for developing progressive symptoms. By contrast, untreated meningiomas which initial diameter was <2.5 cm do not show tumor growth over a follow-up period of 4.6 years. Then, the finding of the parameter 2.5 cm as a threshold for meningiomas *in vivo* allows to describe tumor biologically more aggressive and classify patients who might require or not surgery, which may avoid excessive intervention.

### Spatially-structured models of tumor growth based on partial differential equations

We describe below generic modelling, based on partial differential equations (PDE), which consider the effect that changes in the composition of the medium surrounding the tumors have on their growth. We focus on the study of three key issues: vascular tumor growth, tumor-induced angiogenesis and invasion, and efficacy of treatments such as anti-proliferative and anti-angiogenic therapies. See Appendix B for a more detailed discussion.

The most basic principle for all quantitative models is conservation of mass [49]. Conservation of mass of a component in a dynamic and open system states that:

$${\fontsize{8.1pt}{12pt}\selectfont{ \begin{aligned} {}\left(\begin{array}{l} \text{Net rate of} \\ \textbf{change}\\ \text{of mass} \\ \text{of component} \\ \text{in the system} \end{array} \right) = \left(\begin{array}{l} \text{Mass flow} \\ \text{of the} \\ \text{component} \\ \textbf{into}\\ \text{the system} \end{array} \right) - \left(\begin{array}{l} \text{Mass flow} \\ \text{of the} \\ \text{component} \\ \textbf{out of}\\ \text{the system} \end{array} \right) + \left(\begin{array}{l} \text{Rate of}\\ \textbf{production} \\ \text{of the} \\ \text{component by} \\ \text{transformations} \end{array} \right) - \left(\begin{array}{l} \text{Rate of}\\ \textbf{consumption} \\ \text{of the} \\ \text{component by} \\ \text{transformations} \end{array} \right) \end{aligned}}} $$

The local mass balances are the mathematical form of equality, which can be written as

(3) $$ \frac{\partial C}{ \partial t} = -\nabla \cdot \textbf{J} + r,  $$

where *t* is time; *C* is the concentration of the component in the system; **J** is the mass flux of the component; and *r* is the net production (or growth) rate of the component. This is the *equation of continuity* for a component, either tumor cell densities or chemical concentration of a growth rate limiting (e.g. oxygen or glucose), or of TAF, or of drugs against cancer.

The transport processes that regularly are considered in cancer models are advection (for cell densities) and molecular diffusion (for chemical concentrations). The general expression to model the specific mass flux of a component is

(4) $$ \textbf{J}= \textbf{v} C - D \nabla C,  $$

where **v** is the velocity field due to the mechanical forces which act on the system (e.g. the pressure within the tumor) and *D* is the diffusion coefficient.

We assume that transport of tumor cells is driven only by advection. Consequently, applying the principle of mass balance, the spatiotemporal dynamics of cell populations are formulated as the generic PDE:

(5) $$ \frac{\partial f}{\partial t} = -\nabla \cdot (\textbf{v} f) + r(f),  $$

where *r*(*f*) is the net growth rate of the cell density *f*, which depends on the cell population considered, and $\frac {\partial f}{\partial t}$ stands for the partial derivative with respect to time (representing the rate of change of the mass density *f*). For example, if *f* represents the proliferating tumor cells density, designed as *f*=*P*, then

(6) $$ r(P) = \Gamma P - \delta P.  $$

In replacing *r*(*P*) in (5), one arrives to

(7) $$ \frac{\partial P}{\partial t} + \nabla \cdot (\textbf{v} P) = \Gamma P - \delta P.  $$

This formula is called a *reaction-advection* equation, because of the presence of the advection term ∇·(**v***P*) and the reaction term *ΓP*−*δP*. The term *ΓP* represents the net tumor growth rate, which is determined by local oxygen concentration. For example

(8) $$ \Gamma = \gamma_{0} \frac{1+\tanh(R(C-C_{hyp}))}{2},  $$

where *C* represents the local oxygen concentration, *C*_*hyp*_ is a parameter which describes the sensitivity of cells to hypoxia and *γ*_0_ stands for the maximal proliferation rate. *Γ* is chosen according to (8) in such a way that it satisfies the following properties: *Γ*→*γ*_0_ as *C*≫*C*_*hyp*_, i.e. cells proliferate as the oxygen concentration is high enough, and *Γ*→0 as *C*≪*C*_*hyp*_, i.e. cells do not proliferate as the oxygen concentration is low enough (hypoxia). Moreover *R*>0 is a numerical smoothing parameter, in such a way that *Γ* be a smooth version of a Heaviside function.

The term *δP* represents the necrosis due to a generic anti-proliferative drug, where the parameter *δ* represents the doses of such drug. Eq. 7 is a basic form for modelling tumor proliferation and obviously there are others terms which we could add, for example, a term taking into account the passage to quiescence (see Appendix B).

Similarly, we assume that transport of chemical concentrations is driven only by diffusion. Consequently, applying the principle of mass balance, the spatiotemporal dynamics of chemical concentrations are formulated as the generic PDE:

(9) $$ \frac{\partial g}{\partial t} = \nabla \cdot (D \nabla g) + r(g),  $$

where *r*(*g*) is the reaction term of the chemical concentration *g*. This formula is called *reaction-diffusion* equation. For example, the local oxygen concentration is given by:

(10) $$ \frac{\partial C}{\partial t} - \nabla \cdot (D_{C} \nabla C) = - g_{C} + h_{C},  $$

where *D*_*C*_ stands for the diffusion coefficient of local oxygen in the blood flow, *h*_*C*_ is the production of oxygen in the functional blood vessels, and *g*_*C*_ is the consumption of oxygen mainly due to proliferation tumor cells.

Likewise, the anti-proliferative drug concentration is given by

(11) $$ \frac{\partial M}{\partial t} - \nabla \cdot (D_{M} \nabla M) = - \lambda M - a M P + \rho \delta_{M},  $$

where *D*_*M*_ is the diffusion coefficient of anti-proliferative drug in the blood flow, *λ* is the mean-life of such drug, *a* is the consumption rate due mainly to proliferation tumor cells, *ρ* represents the blood flow, and finally *δ*_*M*_ denotes the doses of the drug administrated at different time intervals. Similar equations also hold for the blood flow *ρ*, the anti-angiogenic drug concentration and the TAF concentration, particularly the VEGF.

Mathematical modelling described above relies on systems of non-linear PDE, in which a set of parameters takes into account the complexity of the underlying biological phenomena. In order to apply such models in practical situations, these parameters need to be identified, that is to say, biologically meaningful values have to be estimated. One way to determine their values is by means of inverse problems theory, exploiting data coming from medical images, as achieved in References [50, 51]. One of the main difficulties is that the amount of data for system identification is scarce. Although medical scans allow an accurate localization of tumors in space, little information can be inferred regarding cellular nature or nutrient distribution inside tumors. In addition, usually only two scans are available before treatment, which makes estimation of tumor evolution a challenging problem.

On the other hand, retrieving tumor shape evolution may provide useful information since PDE based models are spatially distributed. We will return to this important issue in the next section. One possible approach to formulate the inverse problem is by optimal control theory, as was done for instance in [50]. In this approach a PDE-constrained optimization problem has to be solved. For instance, one can consider a functional to be minimized by matching spatiotemporal evolution of tumor density predicted by mathematical modelling with the corresponding tumor density maps estimated from serial scans for one particular patient. In order to carry out the minimization process an adjoint-based algorithm is used for evaluating the gradient of the functional. This algorithm is expensive, since each optimization iteration requires solving a number of forward problems equal to number of variables. A different approach, as was done for instance in [51], consists in using the difference between a variable predicted by the model and its corresponding observed value (the residuals of the model) within a Newton method to solve the inverse problem. This identification procedure is based on proper orthogonal decomposition (POD), a way to identify complex natural processes with models that are intrinsically much simpler than biological scenarios. In this approach the solution is sought in a given low-dimensional functional space, which basis gives an optimal representation of a sufficiently large number of solution samples. In particular, the parameter space is sampled in such a way that all the possible different biological behaviors are represented.

**Fig. 2:**
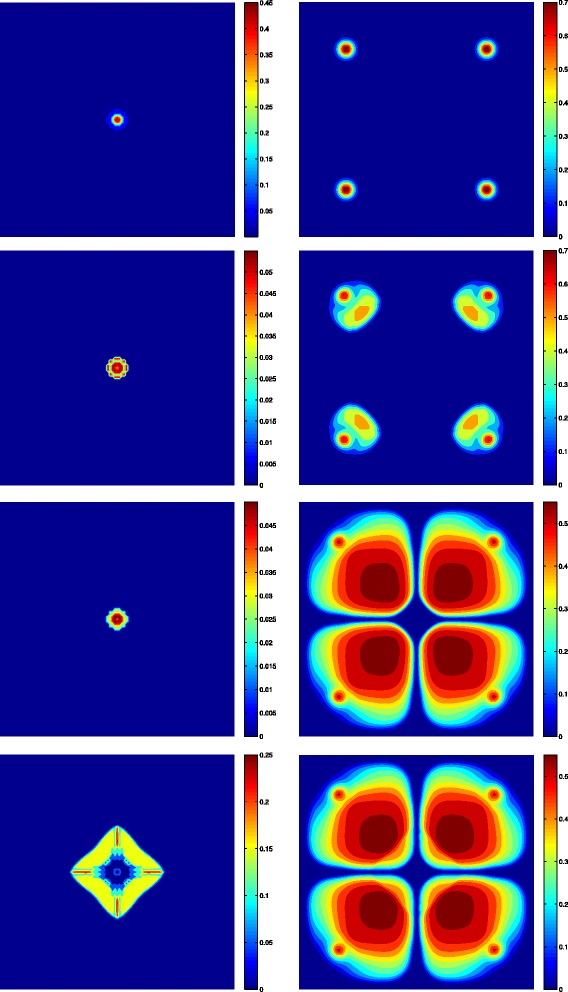
Four snapshots of tumor evolution in absence of treatment at times *t*=10,70,159 and 250 [12 *h*]. (Left) Spatial distribution of proliferative plus quiescent tumor cells. (Right) Spatial distribution of endothelial cells

**Fig. 3:**
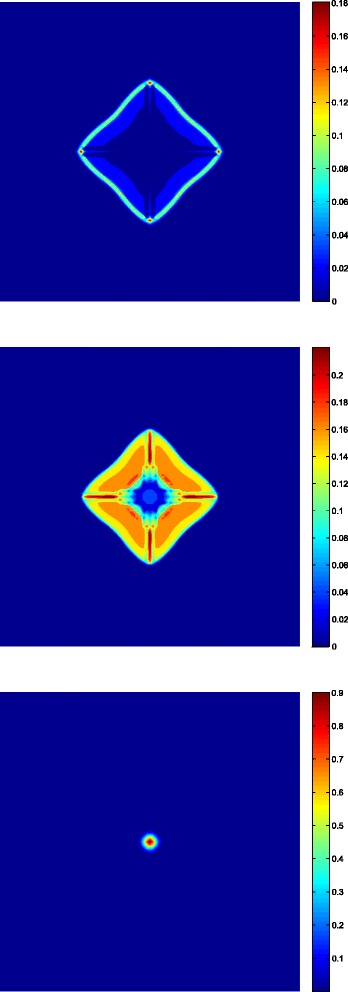
Three layers fully developed distribution of the tumor at time *t*=270[12 h]. Spatial distribution of proliferative cells (up), quiescent cells (middle) and necrotic cells (down)

We finish this section by showing some results which are part of a work in progress. We have carried out some numerical simulations of a tumor growth model under normal conditions, i.e., in absence of treatments. These simulation results are depicted in Fig. 2, which shows the distribution of endothelial cells (right), as well as, the distribution of the sum of the proliferative and quiescent tumor cells (left). The time unit is 12 hours. Because of the configuration (four blood vessels initially placed far from tumor cells), the proliferative and quiescent tumor cells initially decrease, which can be clearly seen in Fig. 2. At the same time, this would produce an increment of the VEGF in the region where the quiescent cells are concentrated. This would trigger the proliferation of endothelial cells making them to move by chemotaxis towards the tumor (see Fig. 2). This process takes place between *t*=30[12 *h*] and *t*=140[12 *h*], generating four sources of nutrients which are enough to increase the oxygen concentration over the hypoxic level. Further, as a result a lack of production in VEGF may be observed which it is thought to be consumed by endothelial cells. At this stage, the main effect is the reactivation of the proliferation of peripheral tumor cells due to high levels of nutrients in a neighborhood of the new blood vessels. However, after some time the oxygen concentration would be consumed by proliferative cells to levels that produce the usual distribution in three layers of tumor cells (see Fig. 3) described in the schedules of fully developed tumors [44]. A diminution in the spread of endothelial cells can also be observed in Fig. 2 as a consequence of tumor growth, which is reflected as a reduction in density of the four blood vessels between *t*=159[12 *h*] and *t*=250[12 *h*]. Accordingly vascular collapse and regression in tumors has been suggested [52], and it is thought that is a consequence of the biomechanical stresses and of the action of the interstitial pressure [53].

## Looking for a hybrid approach

Parameter estimation is highly required in order to improve models for tumor progression and provide new clinical insights. In this regard, we have defined hybrid approach as the feedback capacity between mathematical modelling and biological experimental design. This approach may lead to a better understanding cancer, by fitting mathematical parameters with biological processes, in order to achieve accurate biological predictive models. In this section we will give two examples which illustrate the hybrid approach.

**Fig. 4:**
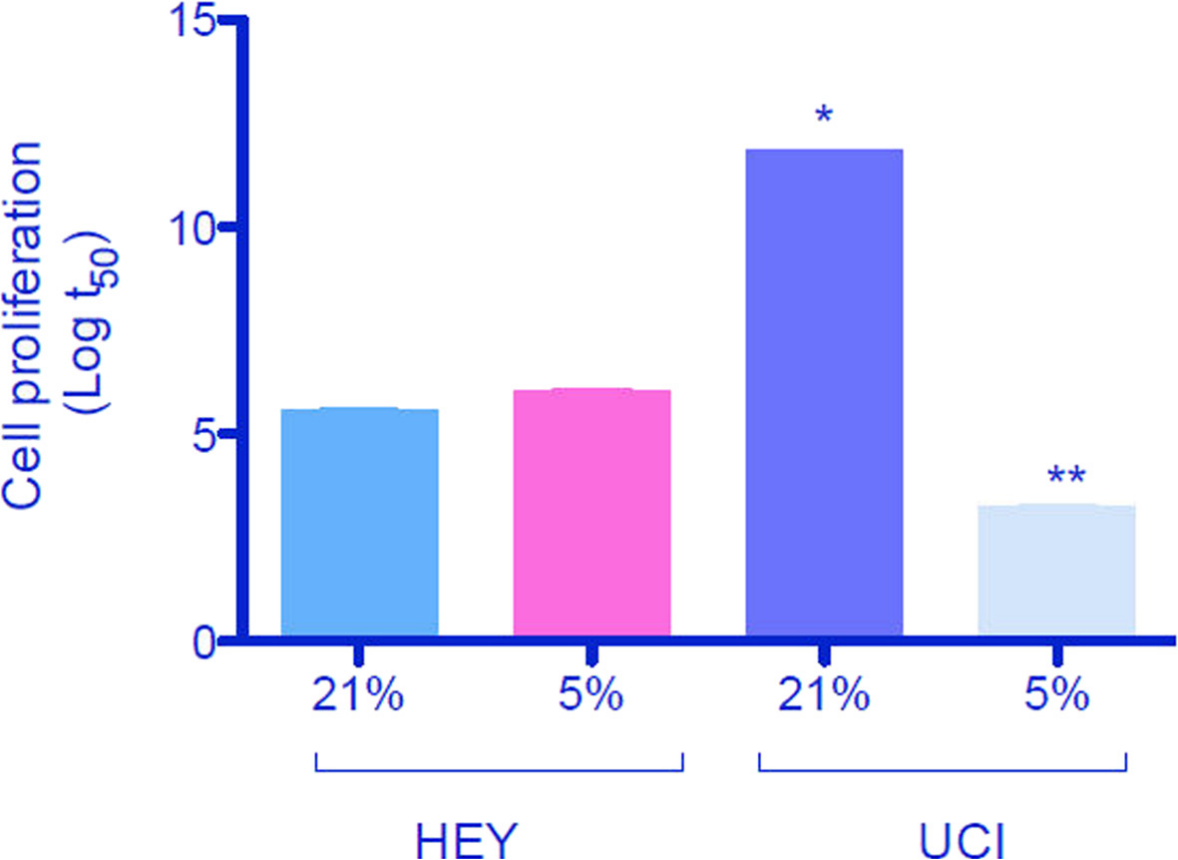
Different cell proliferation in ovarian cancer cells. Two cell lines derived from human ovarian cancers were used (HEY and UCI). Cells were cultured under 21 % and 5 % oxygen during 0, 3, 6, 12 and 24 hours. Cell proliferation was analyzed by bromouridine incorporation as previously reported [58]. Data is presented as the logarithm of *t*_50_ (replication time) ± SEM. *N*=4 per group and analyzed time. * *p*<0.05 vs HEY at 21 % oxygen. ** *p*<0.05 vs UCI at 21 % oxygen

The first example is concerned with tumor cell proliferation. As mentioned in Section Introduction, tumor cell proliferation can be correlated to oxygen concentration, and indeed one could think that the higher the oxygen concentration the higher the tumor cell proliferation. However, this is not always like that. In particular, elevation of anaerobic metabolism in tumor cells during avascular or even vascular tumor growth has been described, a phenomenon called Warburg effect [54], which involves a shift in metabolism away from oxidative phosphorylation (i.e., aerobic) towards anaerobic glycolisis. Therefore, tumor cells are resistant to hypoxia which indeed, depending on the threshold, may stimulate cell proliferation. In this regard, unpublished results from our lab using two ovarian cancer cell lines, HEY and UCI, were cultured under two different oxygen concentrations, 21 % (normoxia) and 5 % oxygen (hypoxia) respectively. Under these conditions, UCI cells were significantly more sensitive to oxygen variations than HEY cells. Indeed under 21 % oxygen UCI cell proliferation was accelerated about 3 times compared to 5 % oxygen, whereas in the case of HEY cells, the proliferation did not significantly change from both different oxygen concentrations (see Fig. 4). Considering these last results into the mathematical modelling described before, net tumor growth rate *Γ* in Eq. 8 need to be remodelled. In Fig. 4, it is clearly seen that UCI cells behave as one would expect, i.e., the higher the oxygen concentration the higher the tumor cell proliferation; in contrast, no matter the oxygen concentration, HEY cells undergo the same proliferation rate. Consequently, whereas net proliferation rate for UCI cells behaves as function *Γ* defined by (8), one should modify the equation for HEY cells. In addition, parameters *C*_*hyp*_ and *γ*_0_ could be estimated using the same experimental setting.

The second example is concerned with tumor drug resistance in gastrointestinal stromal tumors (GIST) liver metastases. In our very recent work [42], we have modelled and simulated resistance to two standard treatments: imatinib and sunitinib. In this regard, the specific tyrosine kinase inhibitors, imatinib, is used as standard first-line treatment. In most of the cases this drug leads to tumor reduction during several months, but then, most of the patients relapse and the tumor is no longer responding to treatment. Then the standard care switches to a second-line treatment using a multi-targeted tyrosine kinase inhibitor (sunitinib), which has both cytotoxic and antiangiogenic effects. After use this second drug, metastasis is controlled for some additional time before a new therapeutical failure occurs. Considering this clinical data, we have splitted the density *P* of proliferative cells (*P*-cells) into 3 subpopulations *P*_1_, *P*_2_ and *P*_3_, such that *P*=*P*_1_+*P*_2_+*P*_3_, where

- *P*_1_ denotes the fraction of proliferative cells that are sensitive to the first-line treatment, based on imatinib molecule, and also to the second-line treatment, based on sunitinib;
- *P*_2_ describes the density of proliferative cells that are resistant to the first-line treatment and sensitive to the second-line treatment; and
- *P*_3_ stands for proliferative cells that are resistant to both treatments.

According to the clinical observations, it seemed relevant to consider that the three cell sub-populations are present when the GIST metastasis is detected. In the mathematical models, we describe tumor growth evolution in terms of tumor area, as well as, its spatial structure and compared with the CT-scans measurements. We believe that intra-tumoral spatial heterogeneity may be related to an increase in the cellular activity, which would mean that a resistant phenotype should be emerging. Indeed according to our modeling, such heterogeneity may be seen as the first stage of the treatment failure, while Response Evaluation Criteria In Solid Tumors (RECIST) [55] do not bring any information about this fact. Therefore our work can be seen as a first step in developing new tools to evaluate tumor response to treatment based on tyrosine kinase inhibitors. Consequently, as mentioned in the previous section, retrieving the evolution of the tumor shape may provide useful information for clinical applications.

According to the discussion above, data coming from clinical observations were crucial in order to develop a complex model that takes into account tumor heterogeneity and treatments resistance. In addition, our model parameters were calibrated in a patient-dependent way, in particular, estimating parameters for two patients and fitted with their specific tumor evolution. Consequently, in this example, mathematical modelling discussed before (in particular Eq. 7 among others) was adapted from cross-talk between physicians and mathematicians based on clinical observations, leading to a new modelling capable to provide better insights for enhancing clinical meaning of mathematical modelling.

## Conclusions and future directions

In this work we described and discussed mathematical modelling of tumor progression, including some aspects as vascular tumor growth, angiogenesis and effectiveness and/or resistance to therapies. We also emphasized what we have called *hybrid approach* in which inverse and progressive confirmation by biological experimentation and use of data from patients with cancer is suggested for enhancing clinical meaning of mathematical modelling. We believe that hybrid approach is a key tool for better understanding cancer, which has been illustrated by two specific examples carry out by our group.

Hybrid approach also involves a big challenge for researchers in cancer field, since despite cancer is deeply analyzed from the particular perspective in each disciplines, when mathematical modelling and biological research face each other, an easier and common language is required in order to go beyond understanding of the pathology. This is not easy to deal with, because there are many differences in the expertise, nomenclature, language and even in the way of thinking that could slow down the communication. Then, one of the main challenges to be overcame is listening and learning each other, maintaining academic discussions where we could bridge the gap on these differences. With hope we have seen a growing number of publications and working groups which are seeking to combine science, but it is still insufficient. In addition, at the same time another step that can be done is the inclusion of physicians in this discussion/analysis in order to look forward potential clinical interest and application of the generated knowledge from basic sciences. These interactions not only will enhance knowledge of cancer, but also improve clinical meaning of mathematical modelling.

Parameter estimation in mathematical modelling may ask very specific questions that need to be designed and tested *in vivo* or *in vitro*. These questions might constitute a challenge for biologist due to limitation in available technology, and also because some parameters are not easy to determine or even not necessarily represent an *in vivo* system, but they are only associated with. For instance, reaction-diffusion Eq. 10 models the balance between variation, diffusion, production and consumption of local oxygen. Despite in mathematical modelling constitutes a usual equation, in biological terms series of experimentation are required in order to analyze how oxygen is delivered towards tumor, how is diffused from vessels to tumor, how tumor cells may control blood flow and oxygen delivery, among others. In particular, future directions should focus in a suitable design of *in vitro* or *in vivo* models for answering such mathematical questions. In this regard, another experimental setting we would like to suggest is isolation of endothelial cells derived from patient’s tissue after biopsies, as it has been already previously developed [56, 57]. This particular cell type might be used for testing specific questions such as how and how much VEGF released from tumor cells may enhance tube formation capacity of that endothelium. Interestingly, anti-angiogenic drugs could also be tested using this experimental setting, which results might be used for parameter estimation.

Future directions in tumor growth modelling should include a cross-talk between biological and theoretical researchers in order to unify effort and generate an active interaction between them. A more common language and understanding each other will be helpful in order to enhance generation of high impact knowledge in tumor growth. In this regard, combination of *in vivo* and *in vitro* experimentation with theoretical analysis is a major challenge which hopefully is changing. In this manuscript we intend to contribute in this field, which of course is limited in comparison with what is needed to be done in order to achieve a better understanding of tumor growth, but it constitutes an example which may alert both mathematicians and clinicians.

## Appendix A: Tumor growth laws based on ordinary differential equations

In this appendix we give some details about of the non spatially-structured models of tumor growth based on ordinary differential equations.

One of the simplest models that can be used to describe the way in which the number of cells *N*(*t*) within a solid tumor changes over time is the exponential growth law which states

(12) $$ \frac{\mathrm{d} N}{\mathrm{d} t} = kN, \qquad \text{with}~~ N(t=0)=N_{0},  $$

whose solution is *N*(*t*)=*N*_0_ exp(*kt*). In Eq. 12, *k*>0 represents the net rate at which the cells proliferate, and *N*_0_ denotes the number of cells initially present within the tumor. In this model, there are no constraints on cell growth: all nutrients and other vital growth factors are assumed to be available in abundance. In consequence, the model predicts that the population will increase exponentially, without limit.

Whilst the exponential growth law is not realistic enough, it provides an accurate description of the early stages of a tumor development. In particular, reduced growth and eventual saturation are observed when avascular tumors are grown *in vitro* or when vascular tumors develop *in vivo*. This discrepancy arises because as the tumor increases in size, competition for nutrients and other vital resources, such as space, can no longer be neglected. A simple modification of (12) which takes account of competition for resources (without specifying what those resources are) is the logistic growth law

(13) $$ \frac{\mathrm{d} N}{\mathrm{d} t} = kN \left(1- \frac N{\theta} \right), \qquad \text{with}~~ N(t=0)=N_{0}.  $$

By using elementary calculus one arrives to

(14) $$ N(t) = \frac{\theta N_{0} }{N_{0} + (\theta - N_{0}) \exp(-kt)} \to \theta \quad \text{as}~~ t \to \infty.  $$

In (13), *θ*>0 represents the carrying capacity of the tumor cells population. Whilst the logistic growth law predicts almost exponential growth of small tumors and growth saturation when the tumor reaches its carrying capacity (*N*=*θ*), the symmetry of *N*(*t*) about its point of inflection (where $\frac {\mathrm {d}^{2} N}{\mathrm {d} t^{2}}=0$ and *N*=*θ*/2) means that it is not particularly flexible to fit or describe experimental data. A more general family of curves, which, depending on the choice of a parameter *α* can saturate more or less fastly than (13), is given by (1), which solution is

(15) $$ N(t) = \theta \left(\frac{N_{0}^{\alpha} }{N_{0}^{\alpha} + (\theta^{\alpha} - N_{0}^{\alpha}) \exp(-kt) } \right)^{1/ \alpha}.  $$

We remark that the logistic growth law is a special case of (1) (set *α*=1) and that the Gompertzian growth law is recovered in the limit as *α*→0^+^. Gompertzian growth law is given by

(16) $$ \frac{\mathrm{d} N}{\mathrm{d} t} = k N \ln \left(\frac {\theta}{N} \right), \qquad \text{with}~~ N(t=0)=N_{0},  $$

whose solution is

(17) $$ N(t)=N_{0} \exp \left(\frac rk (1-\exp(-kt)) \right), \qquad \text{for}~~ r=k \ln \left(\frac {\theta}{N_{0}} \right).  $$

The Gompertzian growth law states that the net proliferation rate of tumor cells exponentially decreases with time. This is apparent because of that

(18) $$ \frac{\frac{\mathrm{d} N}{\mathrm{d} t}(t)}{N(t)} = k \ln \left(\frac {\theta}{N(t)} \right) = r \exp(-kt),  $$

and noting that $k\exp (kt) \ln \left (\frac {\theta }{N(t)} \right) = k \ln \left (\frac {\theta }{N_{0} } \right)$ (i.e. $k\exp (kt) \ln \left (\frac {\theta }{N(t)} \right)$ remains constant in time).

In order to compare the four models presented above, we plot in Fig. 1 growth curves for each model for fixed values of the proliferation rate *k*, the carrying capacity *θ* and the initial condition *N*_0_.

## Appendix B: Tumor growth laws based on partial differential equations

In this appendix we give some details about of the spatially-structured model of tumor growth based on partial differential equations, from which we have obtained the simulation results depicted in Figs. 2 and 3. It is worth noting that this is a work in progress, therefore the previous simulation results are not yet definitive. Moreover, we have not included yet in this model the influence of drugs.

Our model consists in a system of partial differential equations of type advection-diffusion-reaction. This system describes the evolution of the cell densities and of the molecules (oxygen and VEGF), as well as, the interaction between them. The rules that regulate such interactions are, in general, non-linear expressions of the coefficients in the equations. All of these variables undergo temporal dependency (noted by *t*) and spatial dependency (noted by $\vec {x}$). However, to simplify the notation, such a dependence will be omitted and by instance *P* shall be written instead of $P(\vec {x},t)$.

The notations used in the model are shown in Table 2, and the full model proposed for the evolution of tumor growth coupled with tumor angiogenesis is shown in Table 3.

**Table 2:** Notations used in the model

Variable	Description
*Ω*	Computational domain
*P*	Proliferating tumor cells density
*Q*	Quiescent tumor cells density
*N*	Necrotic tumor cells density
*S*	Host cells density
*ρ*	Endothelial cells density
*α*	VEGF concentration
*C*	Oxygen concentration
**v**	Advection velocity
*k*	Permeability of the medium

**Table 3:** Summary of the equations used for the numerical simulation depicted in Figs. 2 and 3

Variable	Equation
Proliferative tumor cells density	*P*_*t*_+∇·(v*P*)=*Γ* *P*+*f*_*QP*_ *Q*−(*f*_*PQ*_+*f*_*PN*_)*P*
Quiescent cells density	*Q*_*t*_+∇·(v*Q*)=*f*_*PQ*_ *P*−(*f*_*QP*_+*f*_*QN*_)*Q*
Necrotic tumor cells density	*N*_*t*_+∇·(v*N*)=*f*_*PN*_ *P*+*f*_*QN*_ *Q*
Host cells density	*S*_*t*_+∇·(v*S*)=0
Endothelial cells density	*ρ*_*t*_+∇·(v*ρ*)+∇·(*g*_*CH*_)=*f*_*ρρ*_ *ρ*
[*O*_2_]	*C*_*t*_−∇·(*D*_*C*_∇*C*)=−*g*_*C*_+*h*_*C*_
[*V* *E* *G* *F*]	*α*_*t*_−∇·(*D*_*α*_∇*α*)=−*g*_*α*_+*h*_*α*_
Advection velocity	v=−*k*∇*φ*
Pressure	−∇·(*k*∇*φ*)=*Γ* *P*+*f*_*ρρ*_ *ρ*−∇·(*g*_*CH*_)

In above equations, *Γ* represents the specific tumor proliferation rate, given by Eq. 8; *f*_*QP*_ the specific rate of transition from the quiescent to the proliferative state; conversely, *f*_*PQ*_ the specific rate of transition from the proliferative to the quiescent state; *f*_*PN*_ and *f*_*QN*_ represent the specific rate of necrosis, from the proliferative and the quiescent state, respectively. These transition functions are given by:

(19) $$\begin{array}{@{}rcl@{}} f_{QP} & = & \gamma_{0} \frac{1+\tanh(R(C-C_{hyp}))}{2}, \end{array} $$

(20) $$\begin{array}{@{}rcl@{}} f_{PQ} & = & \gamma_{0} \left(\frac{1-\tanh(R(C-C_{hyp}))}{2}\right)\left(\frac{1+\tanh(R(C-C_{hyp}^{sev}))} {2}\right), \end{array} $$

(21) $$\begin{array}{@{}rcl@{}} f_{PN} & = & \gamma_{0} \left(\frac{1-\tanh(R(C-C_{hyp}^{sev}))}{2} \right), \end{array} $$

(22) $$\begin{array}{@{}rcl@{}} f_{QN} & = & \gamma_{0} \frac{1-\tanh(R(C-C_{nec}))}{2}, \end{array} $$

where $C_{\textit {hyp}}^{sev}$ is the severe hypoxia threshold, that is to say, the oxygen concentration under which proliferative tumor cells die, and similarly *C*_*nec*_ is the oxygen concentration under which quiescent tumor cells die. One obviously has that $0 < C_{\textit {nec}} < C_{\textit {hyp}}^{sev} < C_{\textit {hyp}}$ to take into account the fact that it is more difficult the quiescent tumor cells die.

The transport of endothelial cells, which are responsible by tumor vascularization, is mainly driven by chemotaxis toward the source of VEGF. This transport term is modelled by

(23) $$ g_{CH} = \chi \, \rho \, \nabla \alpha,  $$

whereas the source of endothelial cells is given by

(24) $$ f_{\rho\rho} = \chi_{prol} \left(\frac{\alpha}{\alpha_{half}+\alpha} \right).  $$

In Eq. 23, *χ* denotes the sensibility to the chemotaxis, which is given by

(25) $$ \chi = \chi_{chemo}\, (1-\rho),  $$

with *χ*_*chemo*_ is the maximum effect of VEGF on endothelial chemotaxis; *χ*_*prol*_ is the maximum effect of VEGF on the proliferation of endothelial cells; *α*_*half*_ is a half-proliferation constant, i.e., it denotes the VEGF concentration such that the proliferation of endothelial cells is exactly half of its maximum (0.5 *χ*_*prol*_).

For the oxygen concentration *C* the source and the consumption are given respectively by:

(26) $$ g_{C}= (\lambda_{C}+\varepsilon_{C} P+\beta_{C} Q) C, \qquad h_{C}= C_{sou} \rho \left(1-\frac{C}{C_{max}}\right),  $$

where *λ*_*C*_ is the oxygen half-life, *ε*_*C*_ is the consumption rate by the proliferating cells, *β*_*C*_ is the consumption rate by the quiescent cells, *C*_*sou*_ the maximum oxygen level available in functional blood vessels and *C*_*max*_ the maximum oxygen level in the host tissue.

In a similar way, the source and consumption terms for the VEGF concentration are given respectively by:

(27) $$ g_{\alpha}= (\lambda_{\alpha}+\pi\rho) \alpha, \qquad h_{\alpha}=\alpha_{sou}Q\left(\frac{1-\tanh(R(C-C_{hyp}))}{2}\right) \left(1-\frac{\alpha}{\alpha_{max}}\right)  $$

where *λ*_*α*_ is the VEGF half-life, *α*_*sou*_ is the source of VEGF located at quiescent tumor cells, *α*_*max*_ the maximum VEGF concentration and *π* denotes the binding rate of VEGF to the receptors of the endothelial cells.

We have assumed that the total number of cells per volume unit is constant (and without loss of generality equal to 1):

(28) $$ P+Q+S+N+\rho = 1.  $$

Adding the equations for the cell densities *P*, *Q*, *S*, *N* and *ρ* (see Table 3), we get:

(29) $$ \nabla\cdot v = \Gamma P - \nabla\cdot(g_{CH})+f_{\rho\rho}\rho.  $$

Thus by imposing the Darcy’s law *v*=−*k*∇*φ* for the advection velocity, where *k* is the permeability of the medium, we deduce the equation for the pressure *φ* (see Table 3).

Finally, we have noted *D*_*j*_ the diffusion coefficients for the different molecules *j*=1,2 (oxygen and VEGF):

(30) $$  D_{j}=D_{j,max} (1-\varepsilon(P+Q+N)),  $$

where *D*_*j*,*max*_ is the maximum diffusion coefficient of the molecule *j* and *ε*>0 is a parameter that represents the percentage of diminution of the diffusion within the tumor, due to the higher density of this last one with respect to the host tissue.

For the boundary conditions, we assumed that the molecules will not go out or in through of the boundary of the computational domain, that is to say, we assumed homogeneous Neumann boundary conditions for *C* and *α*.

For the pressure field *φ* we imposed *φ*|_*∂Ω*_=0. This homogeneous Dirichlet condition is used since we consider that the domain of interest is not isolated, and the outer medium does not impose a pressure on the tumor. This assumption is valid for small tumors that are not mechanically constrained by the extratumoral region.

Finally, Table 4 below shows the parameter values for which simulation depicted in Figs. 2 and 3 was carried out. In Table 4 we use the following notations: h(hour), mm(millimeter), M(molarity).

**Table 4:** Summary of the parameter values used in the simulation depicted in Figs. 2 and 3

Parameter	Description	Value	Units
*C*_*hyp*_	Threshold of hypoxia	5.5	M
$C_{\textit {hyp}}^{sev}$	Threshold of severe hypoxia	1.52	M
*C*_*nec*_	Threshold of necrosis	0.02	M
*D*_*C*,*max*_	Maximum diffusion of oxygen	1	mm ^2^ h ^−1^
*D*_*α*,*max*_	Maximum diffusion of VEGF	0.1875	mm ^2^ h ^−1^
*ε*	Percentage of loss of diffusion	20	%
*C*_*sou*_	Oxygen concentration in functional blood vessels	8	M h ^−1^
*λ*_*C*_	Degradation rate of oxygen	0.01	h ^−1^
*ε*_*C*_	Rate of oxygen consumption by proliferative cells	3	cells ^−1^ mm ^2^ h ^−1^
*β*_*C*_	Rate of oxygen consumption by quiescent cells	1.5	cells ^−1^ mm ^2^ h ^−1^
*C*_*max*_	Maximum concentration of oxygen	8	M
*λ*_*α*_	Degradation rate of VEGF	1.25×10^−4^	h ^−1^
*π*	Binding rate of VEGF to endothelial cells	0.791	cells ^−1^ mm ^2^ h ^−1^
*α*_*sou*_	Production rate of VEGF	2.11	M cells ^−1^ mm ^2^ h ^−1^
*α*_*max*_	Maximum concentration of VEGF	2.11	M
*α*_*half*_	Shape parameter of *f*_*ρρ*_	1.25×10^−3^	M
*χ*_*chemo*_	Maximum effect of VEGF on endothelial chemotaxis	1.25×10^−2^	mm ^2^ M ^−1^ h ^−1^
*χ*_*prol*_	Maximum effect of VEGF on endothelial proliferation	5×10^−2^	h ^−1^
